# An Energy-Efficient Strategy and Secure VM Placement Algorithm in Cloud Computing

**DOI:** 10.1155/2022/5324202

**Published:** 2022-08-25

**Authors:** Devesh Kumar Srivastava, Pradeep Kumar Tiwari, Mayank Srivastava, Babu R. Dawadi

**Affiliations:** ^1^Manipal University Jaipur, Jaipur, India; ^2^Department of ECE, National Institute of Technology, Jamshedpur, Jharkhand, India; ^3^Department of Electronics and Computer Engineering, Faculty of Computer Engineering, Institute of Engineering-Pulchowk Campus, Tribhuvan University, Kirtipur, Nepal

## Abstract

One of the important and challenging tasks in cloud computing is to obtain the usefulness of cloud by implementing several specifications for our needs, to meet the present growing demands, and to minimize energy consumption as much as possible and ensure proper utilization of computing resources. An excellent mapping scheme has been derived which maps virtual machines (VMs) to physical machines (PMs), which is also known as virtual machine (VM) placement, and this needs to be implemented. The tremendous diversity of computing resources, tasks, and virtualization processes in the cloud causes the consolidation method to be more complex, tedious, and problematic. An algorithm for reducing energy use and resource allocation is proposed for implementation in this article. This algorithm was developed with the help of a Cloud System Model, which enables mapping between VMs and PMs and among tasks of VMs. The methodology used in this algorithm also supports lowering the number of PMs that are in an active state and optimizes the total time taken to process a set of tasks (also known as makespan time). Using the CloudSim Simulator tool, we evaluated and assessed the energy consumption and makespan time. The results are compiled and then compared graphically with respect to other existing energy-efficient VM placement algorithms.

## 1. Introduction

In recent years, cloud computing has experienced a meteoric rise in popularity. The latest advances in virtualization technology have elevated it to a significant position currently. Cloud computing has given new life to a wide range of applications, including speech and signal processing [1, 2], thanks to the increased bandwidth and flexibility provided by 5G [3]. A variety of applications are submitted to data centers to obtain pay-per-use services. Because of resource restrictions, on-demand workloads are shifted to other data centers on a regular basis. As a result, workload scheduling in a heterogeneous multicloud environment is a hot topic and a challenging undertaking due to the wide variety of cloud resources available [4–7]. Cloud workloads are planned using VMs, which are assigned based on the amount of traffic and requests. Users may benefit from on-demand, high-quality programs and services from a shared pool of configurable computing resources without having to worry about data storage and upkeep on their home computers [8]. Due to the fact that users with weak computer capabilities no longer have direct access to the outsourced data, data integrity security in cloud computing is difficult for them to maintain. Furthermore, users should be able to use cloud storage as if it were local storage, without having to worry about the integrity of the data stored there.

The application requires a secure infrastructure due to the presence of sensitive data or proprietary procedures [8]. As a result, we treat sensitive requests differently and execute them on a distinct VM. VM placement is an operating mechanism in cloud computing that is implemented to map the most appropriate server or PM to host VM. To improve and enhance the utilization of computing resources, power efficiency, and quality of service (QoS) in cloud computing, choosing the best and most suitable host is crucial and critical. Implementing VM placement in cloud computing is highly difficult, time-consuming, and complex. This is because we cannot predict or forecast how VM starting requests will arrive and study their patterns. Moreover, it is often difficult to accurately find the most suitable and best scenario due to the large size of the data center for the required load.

The data centre resources can be optimised, and energy consumption can be decreased with the use of a suitable VM placement algorithm. This algorithm can manage job scheduling , which helps in reducing the energy usage of data centres. Each VM needs a guaranteed number of computing resources such as memory, CPU, bandwidth, links, and much more to manage the application's security, isolation, and overall performance. Costs can be minimized only when the compute resources are exclusively made available for the application, not based on the load of the work benchmark. To run and maintain all applications' efficiencies, it needs to optimize, monitor, and measure continuously and in real time the data traffic. The data center requires proper precision planning of its network architecture to host hundreds of thousands of devices, including routers, switches, and servers. Until now, lots of advances have been made and have successfully created energy-efficient networking devices and compute servers. Up to 20% of energy conservation can be accomplished by data centers, which also saves up to 30% of the energy requirements that are used for cooling various hardware devices. Currently, a common standard cloud computing deployment model uses quite a large amount of energy, which indirectly produces a large amount of carbon dioxide (CO_2_).

Hence, the goal for cloud providers is to conserve energy as much as possible by implementing highly advanced energy-efficient algorithms, protocols, and top-notch highly advanced techniques. Cloud computing is built to be energy-efficient inherently since it has the features of scalability, meaning it can scale multitenant usage and compute resources automatically. It is also to be noted that low-quality VM placement implemented in data centers results in maximum energy consumption. A Service Level Agreement (SLA) is an agreement that bonds the cloud provider and the customer. This agreement guarantees that at least a minimum level of service is being provided. In VM placement, the allotment of task requested to a set of VMs that are running/computing on several different hosts while following the agreements that contain terms and conditions is declared in SLAs. In this article, we focus on the problem of mapping and allocating VMs to PMs in the data center.

This pertains to decision-making like, when a VM is allocated, which VMs are to be reallocated, which PMs are to be switched off, and which VM is to be allocated to which PM. The VMs are then grouped/clustered based on the type of resources they require. The output is later used as the reference for making decisions for the customized VM instances. These decision-making processes are crucial because they help in saving the energy usage of the entire system by modifying the state of nonactive PMs into disabled mode. The mentioned solution in this article helps in conserving resources and reducing resource wastages in cloud computing system models. This is made possible with the help of virtualization mechanisms and allocating suitable policies for efficiency. We have also formed varieties of subtypes of VMs based on the capability of their resources. The total load of inputs in the data center is calculated by the definite number of input tasks where each individual task is associated with many VMs for execution. During this work, my primary and main aim was to allocate an input task to an already existing VM or else make or create a new VM based on the task and allocate the newly created VM to a currently active host or PM. Because new VMs must be given to the right hosts on a regular basis, there are problems with assigning tasks.

As a result, to deal with the problem of job assignment, we employ the concepts of consolidation and optimal solution. The primary aim is to reduce energy consumption while also appropriately allocating resources in cloud-based applications. To handle this task assignment problem while decreasing energy consumption and ensuring optimal resource allocation in a cloud system, we employ the concept of consolidation and optimum solution approach developed by the authors. Data center time and energy consumption can also be cut down because of the consolidation method.

**Figure 1:**
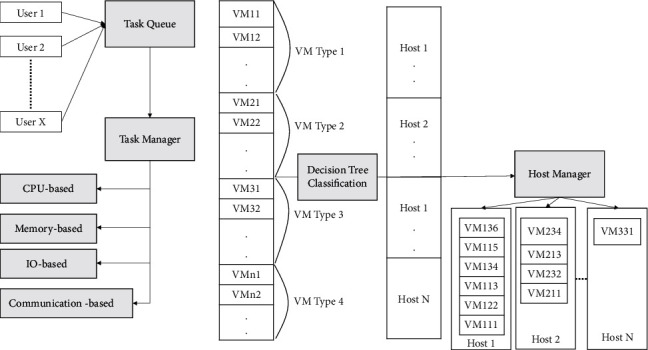
Cloud system model developed.

Consolidation techniques are divided into four subapproaches: server underload detection, server overload detection, VM placement, and VM selection. Each of these subapproaches is discussed in detail below. Consolidation is a tough and time-consuming task; thus, heuristic and meta-heuristic approaches are used to obtain solutions as quickly as possible. The system model proposed in our work is shown in Figure 1, where multiple users can make the request simultaneously.

The proposal made by users will reside in the task queue, which will be allocated to the VM based on the requirements. The critical point here is that, in the IaaS system architecture, the user request can either be a program, task, software, or the operating system itself. The task manager can divide the schedule and tasks and distribute them among multiple VMs to execute the parts of them. In addition, if the request is for system software or an operating system along with a program, then the division of those requests cannot be done in multiple VMs. Therefore, our system model should assign those VMs requests to anywhere in the physical system but not in total allocation. The VM request is being handled by the host as it is requested, depending upon the availability of the resource. The host manager uses a machine learning classifier to ensure the optimal allocation of resources at any moment. We can summarize our contribution as follows:

1. A new cloud system model has been developed in which the input tasks are interpreted as user requests and the cloud computing resources are heterogeneous.
2. To produce a VM Placement Algorithm named “Energy-Efficient VM Placement Algorithm” (EEVMPA) based on task requests, where this algorithm helps in reducing energy consumption, minimizing the rejection rate of tasks, and reducing the makespan time.
3. In cloud computing, various requests are common and frequently made. The tasks can be classified based on their parameters, such as platform, space, priority, type, and sensitivity. We are preparing our data set to build a compatible situation for any given task and its required environments. Based on this, we prepare the decision tree to optimize the allotment. We are using multiple entries, out of which 20% will be used for training and 80% of the data will be used for validation.
4. An overall evaluation of the algorithm is made with the help of the CloudSim tool, and graphical analysis is also done and compared with the other existing energy-efficient algorithms currently available in the market.

## 2. Literature Surveys

Mousa and Hussein proposed an unmanned aerial vehicles (UAVs)-based offloading system; this research goal is reducing the latency and the energy consumption of the UAV. There are two distinct subproblems for the suggested system that are looked into. The suggested offloading method divides the ground devices into clusters, and the UAV passes over each cluster head to perform the tasks that have been offloaded for the members of the cluster. This leads to the first subproblem, which is the clustering of IoT devices, the shortest route for the UAV to travel through the cluster heads [9]. Cloud computing has enormous potential with the advancement of virtualization technologies. Large hardware resources are frequently virtualized. Clients are then assigned to these tiny units. However, these services must be resource-efficient. Many scheduling, allocation, and provisioning issues are framed as optimization issues. Many algorithms were introduced in the past that focused on resource allocation, consumption of energy, and issues faced during scheduling. A detailed literature survey was the need of the hour. Gabhane et al. [10] describe the meta-heuristic model that is extensively studied for VMP in cloud computing. Based on a lot of research and surveys, we found some of the most common related works, like this one. We also looked at their workings, benefits, and drawbacks. Karmakar et al. proposed [11] high-performance computing requires many VMs to meet user demand (VMs). This computation generates a lot of VM-to-VM traffic. Therefore, to satisfy user expectations, VMs must be deployed on actual computers in a way that reduces communication costs and delays. By consolidating VMs into less active physical computers, service providers can reduce operational costs. Finding the best way to reduce deployment or communication costs is NP-Hard. Moreover, focusing on one without the other may result in a cheaper but slower solution (or vice versa). Ant colony optimization is used to manage VM consolidation. The performance of this algorithm was compared with existing mono- and multiobjective algorithms.

Omer et al. [12] focused on large number of complex applications with varying priorities, and resource demands are arising in recent telecommunication paradigms like big data, IoT, UEC, and machine learning. Most researchers use a set of VMs with a set traffic load. Cloud data centers (CDC), a key component of UEC, perform differently depending on how they are placed. VM placement is NP-hard; therefore, no optimal solution exists. This study presents a priority, power, and traffic-aware VM placement strategy in a CDC. A fat-tree topology and the proposed approach were compared. The proposed method decreases total network utilization by 29%, electricity consumption by 18%, and resource waste by 68% compared to the second best findings. Raman et al.'s [13] study is focused on knowing the makespan, and execution cost is required for workflow scheduling efficiency. Optimal workflow scheduling is tough in the cloud since estimating makespan and cost is tricky.

Cloud resources are scheduled to suit user demand. The virtualization approach provides for scalability. This study proposes the PBF-NN hybrid scheduling approach for calculating makespan and execution cost. The backfill algorithm schedules work. This method decreases migration compared to First Come, First Serve. It is then used for resource allocation equity. The system assigns jobs fairly. An energy-efficient VM placement approach is provided by the backpropagation neural network-genetic algorithm (BPGA). By distributing tasks dynamically, it saves resources. Experimental research illustrates how the recommended technique saves money and time. Salami et al. [14] highlight that the VMPP is a frequent provisioning issue. VMPP hosts VM requests on a small number of real computers. The VMPP is solved via Cuckoo Search (CS) and creates new cost and perturbation functions. Using two popular benchmark data sets, Reordered Grouping Genetic Algorithm, Best-Fit Decreasing, and MultiCSA also performed well. Gharehpasha et al. [15] work on store applications, data, and files; cloud computing employs a vast network of systems which reduces power computation, application hosting, content storage, resource waste, and delivery costs. By focusing on corporate goals rather than increasing consumer hardware resources, cloud computing helps organizations achieve their goals. VMs with real equipment in cloud data centers are challenging to place. Cloud data centers may better manage resources by putting VMs ahead of real ones. They also propose a hybrid discrete multiobject whale optimization method with a multiverse optimizer and chaotic function. The suggested method reduces the number of active physical computers in cloud data centers. VMs over real machines in cloud data centers decrease resource consumption. This approach slows the VM to PM migration. A final comparison was made using first fit, virtual machine placement ant colony system (VMPACS) and Modified Best Fit Decreasing (MBFD). Bouhank and Daoudi [16] use placing VMs on appropriate servers in a cloud context for NP-hard issue. They propose a Non-dominated Ranking Biogeography Based Optimization (NRBBO) algorithm to decrease the overall resource waste and power consumption across all servers in a VM installation. To test the approach's efficacy, many experiments were run using generated data from the literature. The findings demonstrate that NRBBO outperforms other multiobjective systems in terms of efficiency, convergence, and coverage.

Mousa and Hussein proposed a UAV-Assisted Computing Mobile-Edge Computing UCMEC system to increase the performance of offloading workloads from mobile devices and reduce task latency and system energy consumption. The suggested method divides the ground devices into regions so that the UAV can fly over each zone and complete the jobs. The partitioning technique employs a VD. Using a Graphics Processing Unit (GPU)-based Particle Swarm Optimization PSO, the UAV's trajectory over the areas is optimised. Authors' algorithm shows the best result among the compared algorithms [17].

Farzaneh and Fatemi's [18] work focused on the critical optimization challenge in cloud architecture that is VM placement (VMP). The solution impacts prices, energy, and performance. PM processing power and VM workloads have powered VMP. The semiconductor industry is also interested in chips having numerous homogeneous or heterogeneous PEs. The newest chip has general-purpose cores with reconfigurable fabric (RF). This study provides a random PE design technique for VMP algorithms. In the given solution, VMP employs RF components in the cloud infrastructure. It's a heuristic to tell the difference. Performance depends on parameter extraction. The obtained parameters are utilized to determine which PM should host the VM. Others outperform our suggested VMP algorithm in our proposed cloud architecture model.

Ghetas [19] proposed a multiobjective Monarch Butterfly Algorithm to manage the VM placement. This study helps to find the location of VMs to place the load.

Gohil et al.'s [20] works on the cloud computing load management is a new way to deliver web services. People, businesses, and academics have all noticed demand-based resource allocation in cloud computing. Rapid data computation and storage expansion in the cloud may produce workload imbalances that breach SLAs and degrade system performance. Workload balancing is crucial in cloud computing. When creating a new VM, the best PM in a cloud data center is chosen. This study examines VM placement in cloud data centers to maximize resource usage. This problem has many solutions. Most available methods balance cloud resources due to inefficient resource consumption. We describe an algorithm that minimizes resource imbalances, waste, and leakage while maximizing resource consumption. Our system selects a host for an incoming VM from a list of hosts based on cosine similarity. The simulation findings show significant gains over existing algorithms like Round-Robin and the default Worst Fit. Chen et al.'s [21] work focuses on reducing energy consumption and thermal costs. It also reduces the number of hot spots in the cloud computing system platform. In this research, Jayanetti et al. proposed a novel hybrid actor-critic technique for resource scheduling in an edge-cloud setting, together with a novel hierarchical state space simulation. The resulting deep reinforcement learning system dramatically decreases the size of the action space handled by each actor network while simultaneously encouraging a distinct separation between edge and cloud nodes, with numerous actor networks directed by a single critic network.

Authors also used proximal policy optimization to get around known issues with conventional actor-critic approaches. In order for the deep reinforcement learning framework agent to develop a balanced trade-off between latency and energy consumption, we also made use of existing works to break down workflow deadlines into individual task deadlines. These were then used as soft upper bounds during the training process. Results from simulation experiments show that the deep reinforcement learning framework works better than all other comparison algorithms by consuming less energy while keeping an algorithm's total execution time that is comparable to other algorithms. [22]. Scheduling problems is proposed by work done by Casas et al. [23] which focuses on the problems of scheduling of scientific workflows in cloud computing systems. Allocation of computing resources is proposed by work done by Hameed et al. [24], which mostly explains how different resource allocations in cloud computing work and gives information on how they work. Kloh et al.'s [25] research work contributes to optimizing running time and lowering costs. This scheduling mechanism is much better than an approach called “join the shortest queue,” but it does not guarantee the best utilization of resources. Deng et al.'s [26] work on a scheduling algorithm for Bag-of-Task (BoT) applications gives us an idea of how scheduling algorithms work in BoT applications. It is a priority based on the tasks and VMs. It then selects the best and most appropriate VM group/cluster for a distinct task cluster. Mosa and Paton's work focused on the management and optimization of energy usage through lower SLA rate violations.

Mosa and Paton developed a self-managing VM placement solution in cloud data centers that dynamically assigns VMs to hosts in accordance with resource use that was provided in this work. It is built on utility functions. The approach's major objective is to maximize an IaaS provider's profit by lowering energy consumption costs and the price of various SLAV sources. The effectiveness of the proposed utility-based approach and an existing heuristic-based solution have been compared through experiments. The proponents of the heuristic method were demonstrated to perform well after applying it to a comprehensive review in comparison to other heuristics.

In both lightly loaded and more heavily loaded cloud data centers, analysis revealed that the suggested utility-based solution beat the current heuristic-based method in terms of energy savings and SLAV minimization [27]. In this paper, we explored the energy consumption optimization of service provisioning within a volunteer cloud. We give a comprehensive solution that includes the service provisioning problem modelling, a hardness analysis, alternative resolution approaches, and experimental examination of those ways. Our investigation demonstrated that we can find effective algorithms for the real-time optimization of our service supply [28]. Belgacem et al. proposed an Intelligent Multi-Agent Reinforcement Model (IMARM) for optimizing cloud resource distribution in cloud. It provides a comprehensive solution for cloud service providers since it addresses the problem of resource allocation from several angles.

Using mechanisms for checkpointing and VM migration, the suggested approach achieves load balancing and provides fault tolerance. Sensor agents transmit system failure status, energy use, and workload on a periodic basis to track system reaction (recovery, downtime, migration, etc.). Q-Learning enables the indication of the action to be taken in each system state, recognizing that the weight of the virtual machines, overall energy consumption, and quantum time are the major factors employed to optimize the system. With the option to choose whether a task or virtual machine should be moved, restored, or shut down, it offers a fascinating new approach for determining the optimal response to shifting cloud settings. The outcomes and experimental analyses of proposed algorithm work better than other methods. By providing a minimal execution time while utilising resources effectively, it demonstrated a positive influence on the execution time [29]. Work of Mastroianni et al.'s [30] research is based on the idea of Bernoulli trials, and it works on two main principles: self-organization and adaptability. This makes it more efficient at managing large data centers.

## 3. Materials and Methods

Based on SLA policies agreed upon between the cloud provider and users, VM placement can dynamically map VMs onto PMs/hosts. Inside the PM/host, VMs are controlled and managed by a specific layer, which is a software known as the Hypervisor. There are two types of VM placement schemes. Those are as follows: (i) Static VM Placement: this is a VM mapping methodology in which the mapping technique is fixed and cannot be recomputed for an extended period of time. (ii) Dynamic VM Placement: it is a mapping methodology of VMs in which the initial placement can be changed because of some changes (dynamics) that occur during the system computing load or other types of loads. A Dynamic VM with a placement mechanism is used in this research due to its flexibility during execution and its ability to minimize energy consumption. To create the energy-efficient algorithm, we first developed a cloud system model, which is a scheduling model where the tasks are advanced through various entities, such as task queue, task manager, task segregator, various VM-type allocation, host manager, and various hosts. These are all included in a cloud data center. The cloud computing system has *m* hosts (*H* = {*H*1, *H*2,…, *Hm*) which are heterogeneous in nature with respect to the capacity of resources. Initially, tasks are received from the users over the Internet. These tasks are known as “service requests.” A cloud service provider (CSP) provides these services to users when they request any service. Users present their tasks to the task queue, and the task manager separates and clusters the tasks into four main, uniquely different categories of tasks. These four categories are CPU-based, memory-based, I/O-based, and communication-based. These tasks are separated into four types because it could map similar types of tasks to VMs quite easily. Hence, this methodology is implemented. The task manager later gets to know about the types of VMs and learns about the subtypes of VMs that are offered. As a result, four types of VMs are created to map smoothly with the VM. They are named as VM1 for CPU-based tasks, VM2 for memory-based tasks, VM3 for I/O-based tasks, and VM4 for communication-based tasks. Each VM-type further contains a subtype (VMab), which represents *a*th VM of *b*th type). After mapping the tasks into the VM-types, the four VM-types are submitted to the host manager, where the host manager gets to study the whole information about the VM-types and record it. The host manager later chooses the appropriate VM type based on the task type to be processed and then creates a suitable VM (VM) on the PM (PM) or host. By doing this, energy consumption can be minimized and makespan time can also be reduced significantly. At the start, all hosts are in an inactive or sleep state. The host manager checks if any active host has adequate resources to create a VM. If that condition is satisfied, then it creates a VM on that active host. This methodology helps in saving energy. If there are no active hosts, then the host manager goes towards a nonactive/sleep host, changes its mode status to an active state, and then creates a brand-new VM on that host. When there is a situation where no tasks are present in the task queue, then the connection between the VM and the PM host is terminated, and the resources are set free from usage.

A specific energy model is adopted throughout this project/research, which helps in reducing energy consumption. In this model, energy consumed by a task can be calculated. Ultimately calculating the total energy consumed by individual tasks results in finding out the overall energy consumption of the whole data center. Figure 1 shows the host manager managing the available hosts, and these hosts contain the serial number of VMs. Each host is defined by a unique ID to identify the host, and each host VM also has a unique ID to identify the VMs. It helps to manage the load on different hosts. The task manager manages the tasks of different userbases, and these userbases are also identified by a unique ID. A unique ID helps to manage the requested resources and capacity of infrastructure that are already assigned to a particular userbase.

Energy consumed by a task (*t*_*x*_) is represented as *E*_*xyz*_, where energy is consumed by *x*th task which is present on *y*th VM (VM) and *z*th PM (PM) or host. The formula for calculating *E*_*xyz*_ is as follows:

(1) $$\begin{array}{c} E_{\text{XY}z}=E_{xyz}^{tr}+E_{xyz}^{\mathrm{Pr}}, \end{array}$$

where *E*_*xyz*_^*tr*^ is the energy consumed during file data transfer and *E*_*xyz*_^*pr*^ is the energy consumed during task execution.

Energy consumed during file data transfer, i.e., *E*_*xyz*_^*tr*^, is calculated by the following formula:

(2) $$\begin{array}{c} E_{xyz}^{tr}=\left\{\sum_{l=1}^{h}T_{i}metr\left(f_{xYz}^{l}\right)\right\}\times P_{\text{avg}}, \end{array}$$

where *E*_*xyz*_^*tr*^ is calculated by the product sum of all needed files transfer time with the transfer of data's average power consumption (*P*_avg_).

The host's average power consumption (*P*_avg_) in the data center is calculated as follows:

(3) $$\begin{array}{c} P_{\text{avg}}=\left(\sum_{z=1}^{m}{\text{Pactive}}_{z}\right), \end{array}$$

where *P* (active) is the power consumed by the *z*th host which is in active state.

Energy consumed during task execution, i.e., *E*_*xyz*_^*pr*^, is calculated by the following formula:

(4) $$\begin{array}{c} E_{xyz}^{\mathrm{Pr}}={\text{ET}}_{xyz}^{I}=P_{yz}^{I}, \end{array}$$

where *E*_*xyz*_^*pr*^ is calculated by the product of task execution time (ET_*xyz*_^*I*^) and the average power consumed by *y*th VM (I-type) of *z*th host. I is the VM type where *I* can have four values, i.e., (*I* = {1, 2, 3, 4}). If *I* = 1, then it is CPU-based tasks. If *I* = 2, then it is memory-based tasks. If *I* = 3, then it is I/O-based tasks. If *I* = 4, then it is communication-based tasks.

(5) $$\begin{array}{c} {\text{NETC}}_{ijk}=\frac{\left({\text{ETC}}_{ijk}-{\text{Minimum}}_{ij}\right)}{{\text{Maximum}}_{ij}-{\text{Minimum}}_{ij}}. \end{array}$$

(6) $$\begin{array}{c} f\left(x\right)=\left\{\begin{array}{cc} \text{MCXXS}\left(i;m2+1\right); & if\;m=\text{odd,}\\ \text{MCXXS}\left(i;m2\right)+\text{MCXXS}\left(i;m2+1\right)2; & \text{otherwise.} \end{array}\right. \end{array}$$

Some of the important tools such as CloudsSIM 3.0.3 and NetBeans 7.4(IDE) are used for the analysis and simulation: the median to perform the task over cloud can be calculated using the below equation. The normalized ETC matrix element NETC*ij*; *k* is formed by taking the ratio between the difference in ETC*ij*; *k* and the minimum execution time of task *Ti*; *j* and the difference in maximum and minimum execution time of task *Ti*; *j*.

## 4. Problem Statement and Formulation

VM Placement plays a crucial and important role in the cloud computing data center. It helps in a large way to reduce energy consumption and enables proper resource utilization in the data center. Here in this project/research, we have considered *m* hosts, which are in a heterogeneous state. Each host is either in an active or inactive (sleep) state at the beginning of the stage. There are also *k* types of VMs in this cloud system that are classified based on their resources. By using the same idea for VM types, tasks are also categorized into the same task clusters so that they can fit into one suitable type of VM. Hence, the task manager plays a crucial role in this algorithm as it gets complete information about the service request tasks from the task queue, categorizes these tasks into four main task groups, and uses this information to decide whether to create new VMs or not in the host/PM. Therefore, due to this circumstance, a job assignment problem arises as it constantly deals with the allocation of newly created VMs to appropriate hosts. Hence, we implement the idea of the consolidation method and optimal solution methodology to solve this job assignment problem. However, our prime focus is to reduce energy consumption and proper resource allocation in cloud systems.

Now implement the idea/concept of the consolidation method and optimal solution methodology to solve this job assignment problem while keeping our primary focus on reducing energy consumption and proper resource allocation in the cloud system. Algorithm also helps us to make span time and energy consumption-aware in the data center. The consolidation method is a complicated approach which can be further divided into four subapproaches, those are server underload detection, server overload detection, VM placement, and VM selection. Finding solutions using the consolidation method is difficult and time-consuming. Hence, heuristic-based and meta-heuristic-based approaches are used to find solutions quickly and efficiently.

## 5. Methodology

The following are the methods implemented during the research work: (i) a new cloud system model is developed where the input tasks are taken as the requests from the users and the cloud computing resources are in a heterogeneous state. (ii) A VM Placement Algorithm named Energy Efficient VM Placement Algorithm (EEVMPA) is produced based on task requests, where this algorithm helps in reducing energy consumption, minimizes the rejection rate of tasks, and reduces the makespan time. (iii) In cloud computing, heterogeneous requests are common and requested frequently. The tasks can be classified based on their parameters, such as the platform, space, priority, type, and sensitivity. We are preparing our data set to build a compatible situation for any given task and required environments. Based on this, we prepare the decision tree to optimize the allotment. We are using multiple entries, out of which 20% will be used for training and 80% of the data will be used for validation. (iv) An overall evaluation is made on the algorithm with the help of the CloudSim tool, and graphical analysis is also done and compared with the other existing energy-efficient algorithms currently available in the market.

## 6. Proposed Work

In this research work, we developed seven algorithms, where one of the algorithms is then in the form of MB (megabyte). IOx is the *x*th task's input/output needs. *b*_*x*_ is the bandwidth of the *x*th task and is in the form of MB (megabytes). The EEVMPA consolidation algorithm is Algorithm 1 where tasks set (TS) with *n* number of tasks, deadline set (DLS) with a *n* number of deadlines, hosts set (HS) with a *m* number of hosts, and VM-types (VMt) with four types, i.e., {*y* = 1, 2, 3, 4}, are given as input resources to the algorithm. This algorithm gives us output as Algorithm: in Algorithm 2, in Step 2, another subalgorithm is called “CategorizedTask”—Algorithm 3, which helps to categorize all the tasks into the mentioned four categories. Here the sorted, updated, and refreshed task queue RQ is given as input. Here, the task resources, which are made into five tuples, are used and represented as RPTK_*x*_, which should also contain a lower bound and upper bound for each tuple. RPTKx = {Len_*x*_, dls_*x*_, MM_*x*_, IO_*x*_, b_*x*_}. CPUB is the upper bound and CPLB is the lower bound for task lengths. DLSU is the upper bound and DLSL is the lower bound for tasks' deadline. MMU is the upper bound and MML is the lower bound for tasks in main memory. IOU is the upper bound and IOL is the lower bound for task IO requirements. bU is the upper bound and bL is the lower bound for task bandwidth.

Algorithm 3 returns the four categorized tasks sets, i.e., CPU-based, memory-based, I/O-based, and communication-based. In Step 1 of Algorithm 3, processing speed's upper bound (PSU) is calculated from Step 2 to Step 14 in this algorithm, a loop is created and at the *x*th iteration, *x*th task is deployed at one of the four categories for the tasks. In Algorithm 1, we could notice that in Step 1, a subalgorithm is called (SortedQueueTask—Algorithm 2) with the help of set of tasks and deadlines. Algorithm 2 helps in sorting all the service request tasks in ascending order based on their deadlines. Algorithm 2 consists of Delete Min function which helps in deleting the deadlines, where *r* is of minimum value from the set of tasks, and then stores it in the RQ (Refreshed Queue) mentioned to calculate the values of vc_*x*_, vm_*x*_, vio_*x*_, and vb_*x*_, which have the value between 0 and 1. But when you add all these, then the sum should be equal to 1.

In Step 6, we could see that all the values of vc_*x*_, vm_*x*_, vio_*x*_, and vb_*x*_ are multiplied with *z*_*x*_. After multiplying, we compare all the four values and find out the Max value which determines the category of the task. In Algorithm 1, from Step 3 to Step 9, the loop runs *x* number of tasks. In Algorithm 4—VMsFree, the VMs which are in free/no work state change to sleep or inactive mode/state and those VMs compute resources are transferred to the next host. The active host connected with its allotted VM is provided as input to this algorithm.

The ultimate goal of this algorithm is to reduce the number of active VMs and regularly keep them updated with the host manager. In Algorithm 1, Algorithm 4 is a subalgorithm for it, and it is called. In Algorithm 5—Hosts Free, it is a subalgorithm for Algorithm 1 where it is called at Step 5 in it. This algorithm gets input as an active host set and an active VM set. Steps 1 through 5 of the loop assist in converting the host's state, i.e., it converts an active host into a sleep or inactive state if the host is not working or is in an idle state. The state of the host will be changed from Step 7 to Step 18. If VMs on a minimum loaded host/PM can be fully migrated to other hosts that are in an active state, then all the VMs are migrated to other active hosts and the previous host changes its mode to sleep or inactive state.

In Algorithm 1, in Step 6, a subalgorithm is called (Algorithm 6—VMTypeSelection). Algorithm 6 chooses a VM-type based on the task type and the requirements of the tasks. Here, tasks, task types, VM types, and VM subtypes are sent as input. VM types are of four types, that is VM type1 for CPU-based, VM type2 for memory-based, VM type3 for I/O- based, and VM type4 for communication-based. There are *n* number of VM subtypes in a VM type. For example, for VM type1, the VM subtypes are as follows: in Algorithm 1, in Step 7, a subalgorithm is called (Algorithm 7—host selection).  Algorithm 7 helps in finding the best host for the specific VM type. The VM type that was found from Algorithm 6 is used as input here along with the hosts which are set in active state and the hosts which are set in a sleep/inactive state. In this algorithm, steps 1 to 6 help us find an active host so that a VM can be placed on it. In steps 7 to 13, this loop helps in searching for sleep state hosts if any other active hosts are not available. Using the heuristic and meta-heuristic consolidation methodologies here, we can achieve our main aim of finding the best active host for the VM.

## 7. Results and Discussion

**Figure 2:**
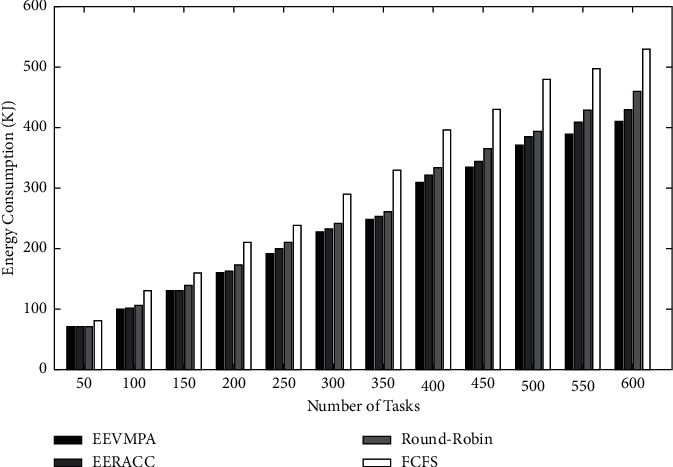
Energy consumption for EEVMPA, FCFS, Round-Robin, and EERACC.

After developing all the algorithm modules, these modules are transferred and performed in the CloudSim 3.03 simulator. Using the CloudSim Simulator tool, we evaluated and assessed the energy consumption and makespan time. The results are compiled and then compared graphically with respect to other existing energy-efficient VM placement algorithms. We also used a software called Xen, which is used as an operating system (Hypervisor) which constantly monitors the VM. This developed algorithm (EEVMPA) is implemented in the Java language. NetBeans 1.7 (IDE) and JDK 1.7 software are also used to provide a platform for the cloud simulator. This experiment was tested on a HP workstation which has an Intel i3 1.19 GHz CPU and 12 GB of memory. During simulations, several cloud computing resources are used, such as user request tasks, VMs, and hosts that are heterogeneous in nature. The length of service requests by users and the requirement of resources are randomly created. During simulation, for every test case, the number of hosts is kept fixed, and the number of VMs (VMs) is kept in the range of 10 to 200. The VM resources are randomly generated and should satisfy the condition that these resources should be less than the capacity of the host on which they are deployed. In terms of calculating performance, we compare all VMs' energy consumption with respect to the entire server's energy consumption one by one. To avoid anomalies, 10 different runs of the entire algorithms for the specific user service request tasks are implemented. In this experiment, we have used four different ranges for the length of tasks. The ranges are as follows: (3500–5500), (6500–8500), (8500–10,000), and (10,000–11,500). Each and every input task in the set contains 25% of the tasks from any one of the ranges of the task length mentioned before. In this experiment, we compare the developed EEVMPA with the other existing VM placement algorithms. Those algorithms are (1) FCFS (First Come, First Serve) Algorithm, (2) Round-Robin Algorithm, and (3) Energy-Efficient Resource Allocation (EERACC Algorithm). Then, many graphical comparative analyses (bar-graph representation) of these algorithms, including EEVMPA, is made. In terms of energy consumption in these algorithms, we implemented graphical analysis based on two different conditions. In condition 1, we have compared all four algorithms when the number of VMs is fixed, and the number of tasks is varied.  Figure 2 shows the graphical comparison of energy consumption for EEVMPA, FCFS, Round-Robin, and EERACC when the number of tasks is varied, and the number of VMs is fixed. From the figure, we can see that EEVMPA consumes less energy as compared to the rest of the VM placement algorithms when the number of tasks increases, and the number of VMs is kept fixed. In condition 2 in terms of energy consumption, we have compared all four algorithms when the number of VMs is varied, and the number of tasks is fixed.

**Figure 3:**
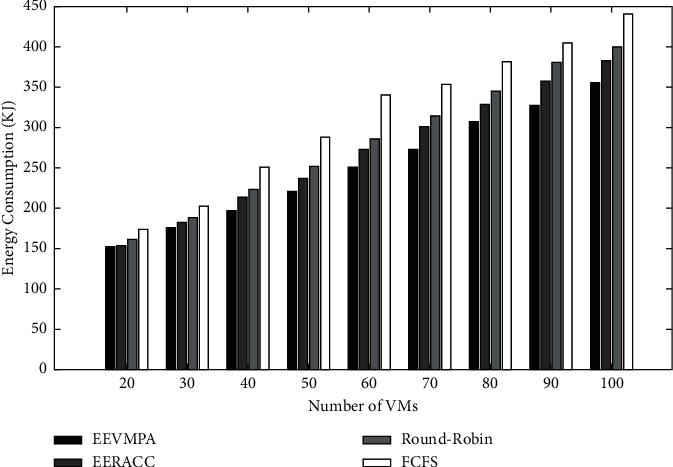
Energy consumption for EEVMPA, FCFS, Round-Robin, and EERACC.

The graphical analysis of this condition is shown below. From Figure 3, we can see that when the number of tasks is fixed and the number of VMs is varied, EEVMPA consumes less energy than the other VM placement algorithms. In terms of makespan time in these algorithms, we implemented graphical analysis based on two different conditions. In condition 1, we have compared all four algorithms when the number of VMs is fixed, and the number of tasks is varied.

Later, this situation is solved as the number of tasks increases, the EEVMPA is able to adapt and then provides less makespan time compared to other algorithms. This is especially true when the number of tasks is varied, and the number of VMs is fixed. In condition 2, in terms of makespan time, we have compared all four algorithms when the number of VMs is varied, and the number of tasks is fixed. Figure 3 depicts the comparison of energy consumption for EEVMPA, FCFS, Round-Robin, and EERACC when the number of tasks is fixed and the number of VMs is varied.

EEVMPA initially performs worse compared to other algorithms as it takes maximum makespan time. This is because EEVMPA attempts to use the minimum number of hosts possible, but in general, cloud systems take a greater number of tasks, so this situation occurs.

**Figure 4:**
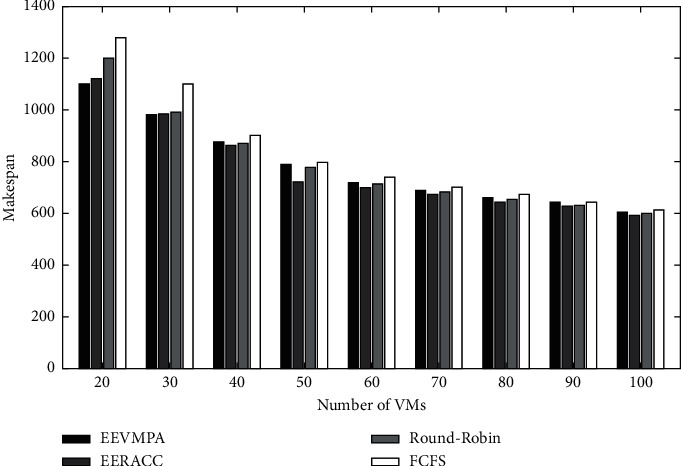
Makespan time for EEVMPA, FCFS, Round-Robin, and EERACC.

**Figure 5:**
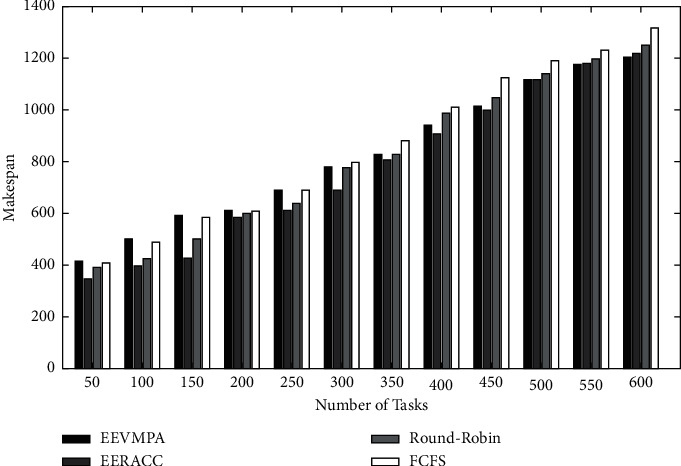
Makespan time for EEVMPA, FCFS, Round-Robin, and EERACC.

Figures 4 and 5 depict the graphical comparison of makespan time for EEVMPA, FCFS, Round-Robin, and EERACC when the number of tasks is varied and the number of VMs is fixed. From Figure 5, we can see that at first, EEVMPA takes less time to make and performs better than other algorithms we compared. As the number of VMs grows, all the algorithms come to the same level of makespan. This is because the longer the VMs work, the performance slows down significantly, and hence, this result occurs when the number of tasks is fixed but the number of VMs is varied.

## 8. Conclusions

The main aim of this algorithm is to minimize energy consumption and reduce makespan time, which is crucial for getting the best performance from the data center. Both targets were successfully achieved in this work. In this research, we compared EEVMPA with other existing VM placement algorithms such as the First Come, First Serve (FCFS) algorithm, the Round-Robin algorithm, and the Energy Efficient Resource Allocation (EERACC) algorithm and did graphical analysis among them to determine which algorithm performed the best in each condition. Our method is found to be the best in economizing energy consumption, related power costs, and reducing makespan time.

## Figures and Tables

**Algorithm 1:**
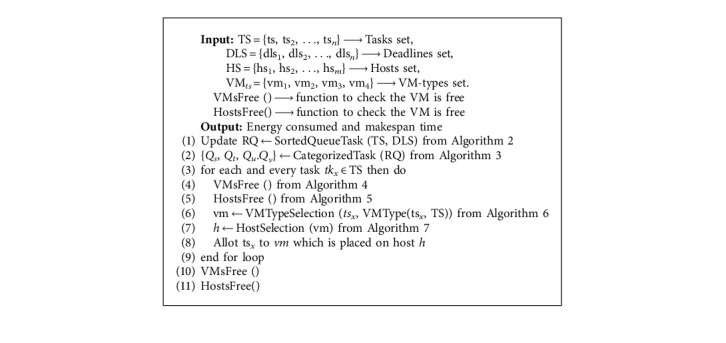
Energy efficient VM algorithm (EEVMPA).

**Algorithm 2:**
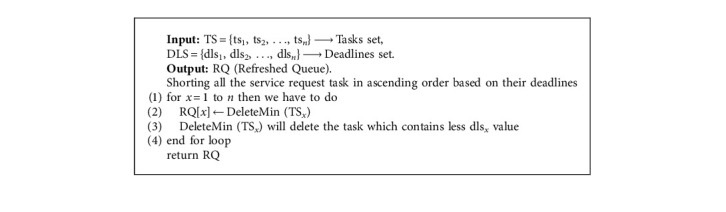
Sorted queue task.

**Algorithm 3:**
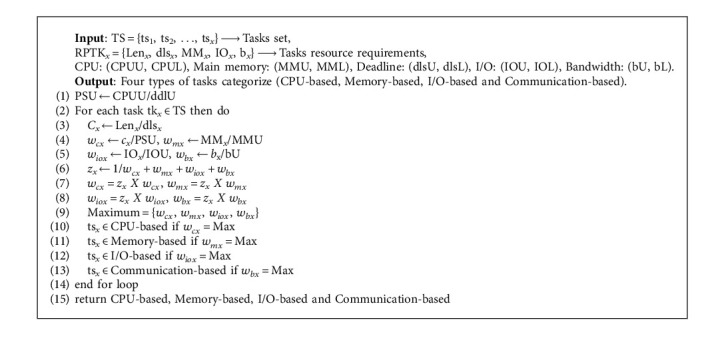
Categorized task.

**Algorithm 4:**
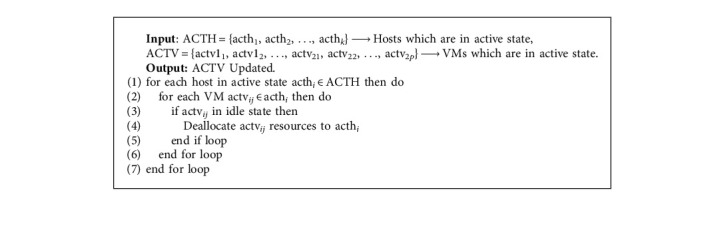
VMs free.

**Algorithm 5:**
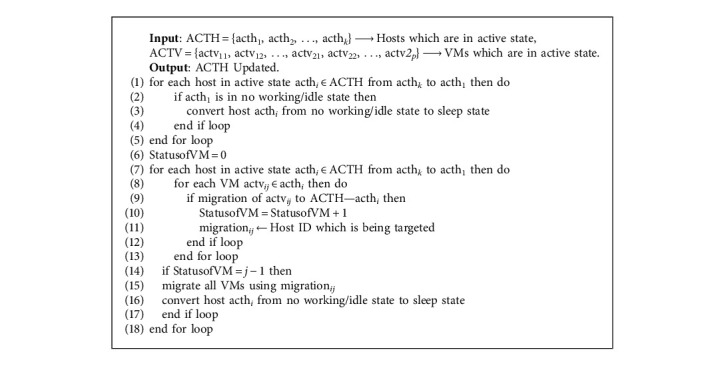
Hosts free.

**Algorithm 6:**
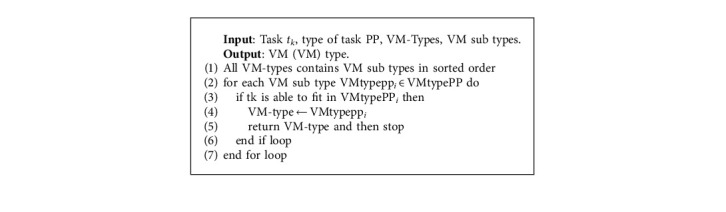
VM type selection.

**Algorithm 7:**
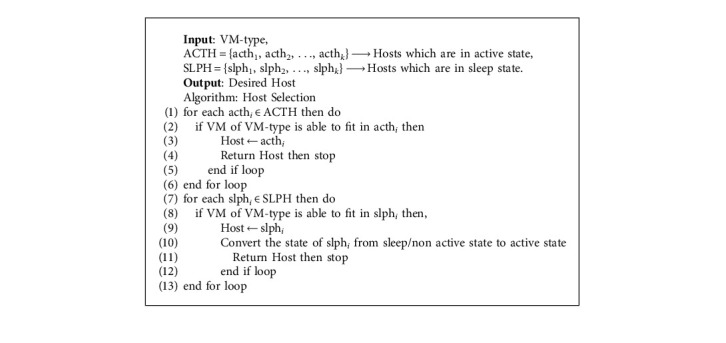
Host selection.

## Data Availability

The manuscript contains all the data used in the study.
